# One-Week Maternal Separation Caused Sex-Specific Changes in Behavior and Hippocampal Metabolomics of Offspring Rats

**DOI:** 10.3390/brainsci14121275

**Published:** 2024-12-18

**Authors:** Meng-Chen Dong, Yu-Xin Chen, Xin-Ran Sun, Ning Jiang, Qi Chang, Xin-Min Liu, Rui-Le Pan

**Affiliations:** 1 Institute of Medicinal Plant Development, Chinese Academy of Medical Sciences and Peking Union Medical College, No. 151, Malianwa North Road, Haidian District, Beijing 100193, China; mcdong@student.pumc.edu.cn (M.-C.D.); chenyuxin@implad.ac.cn (Y.-X.C.); sunxinran@student.pumc.edu.cn (X.-R.S.); njiang@implad.ac.cn (N.J.); qchang@implad.ac.cn (Q.C.); 2 Institute of Drug Discovery Technology, Ningbo University, No. 818, Fenghua Road, Jiangbei District, Ningbo 315000, China

**Keywords:** maternal separation, behavioral tests, hippocampus, untargeted metabolomics

## Abstract

To investigate the effects of one-week maternal separation (MS) on anxiety- and depression-like behaviors in adolescent and adulthood as well as adult hippocampal metabolomics simultaneously in offspring female and male rats. In the MS group, newborn SD rats were separated from their mothers for 3 h per day from postnatal days (PND) 2 to 8. The open field test (OFT), elevated plus mazes (EPM), novelty suppressed feeding test (NSFT), and forced swimming test (FST) were conducted during adolescence and adulthood. Serum corticosterone, mRNA expression of hippocampal inflammatory cytokines, and hippocampal untargeted metabolomics of offspring adult rats were examined using an assay kit, qRT-PCR, and UPLC-Q-TOF/MS. Both MS female and male rats showed similar behaviors in OFT, EPM, NSFT, and SPT, except for the latency to feeding during adolescence and the open arm entries during adulthood, showed statistical significance only in MS female rats. Serum corticosterone and hippocampal pro-inflammatory cytokines IFN-γ were significantly elevated in both female and male rats, and IL-1β and TNF-α were significantly increased only in female rats. In hippocampal metabolism, the identification of differential metabolites displayed 53 and 37 in female rats and male rats, respectively (with 35 common metabolites), which were involved in 33 and 30 metabolic pathways with 28 common pathways. One-week MS induced sex-specific anxiety- and depression-like behaviors in female and male offspring rats during adolescence and adulthood, as well as sex-differentiated characteristics in the hippocampus inflammatory cytokines and metabolomics of adult MS rats. From the experimental data, the effects of MS on the female offspring rats were more severe than those of the male offspring rats.

## 1. Introduction

Life in the early stage is a sensitive period of rapid brain development and physiological growth. If this stage encounters adverse stimuli, such as maternal neglect, maltreatment, mental stress, and disease, there will be long-term negative effects on physical and mental health. Studies have shown that children with prolonged separation from parents suffered cognitive impairment, behavioral abnormalities, and other neuropsychiatric disorders in adulthood [1].

Since maternal separation (MS) in rodents is similar to the stress of human adversities encountered in the early stages of life, it was often used to induce animal models, such as anxiety and depression [2,3]. Through a large-scale literature analysis, it is found that MS is mainly carried out in two forms, concluding maternal deprivation, which is carried out on a certain day of the lactation period for 24 h stress, and continuous maternal separation, a main MS paradigm, which is carried out for a certain period of time (usually 3–4 h) every day during the lactation period. Moreover, it is also found that published studies on MS commonly used pregnant rats, generally researched male offspring rats, had a start time that was usually set on postnatal day (PND) 0–3, most often PND2, and the end time was set at PND14 [4,5] or PND21 [6,7]. Additionally, behavioral testing is usually performed in adulthood in previous studies. Above all, the effects of MS one week after birth on the anxiety- and depression-like behaviors during adolescence and adulthood of male and female offspring rats were less reported.

The hippocampus is a highly sensitive brain region that mediates stress responses in rodents, and there have been many reports on the hippocampus of rodents after MS, which showed that MS affects hippocampal development and results in decreased proliferation; slowed differentiation and increased apoptosis of hippocampal neurons; reduced neuronal plasticity and dendritic complexity; caused hippocampal neuroinflammation and increased pro-inflammatory factors [2,8,9]. However, few studies have been reported on the hippocampal metabolism of rodents after MS.

Since the postnatal period is most critical for mother–pup interaction [10,11], MS for the first week after birth, which means MS from PND2 to 8 of SD rat pups, and 3 h for separation every day was selected in this study. With a view to comparing the differences in behavior and hippocampal metabolism in MS female and male offspring rats, behavioral tests, including open field test (OFT), elevated plus mazes (EPM), a novelty suppressed feeding test (NSFT), and a forced swimming test (FST), were conducted during adolescence and adulthood, respectively; serum corticosterone, inflammatory cytokines, and the untargeted metabolomics of the hippocampus were examined. This study provides a scientific basis for studying the mechanisms of MS effects on offspring rats and developing related healthcare drugs.

## 2. Materials and Methods

### 2.1. Experimental Material

Acetonitrile and methanol for mass spectrometry were purchased from Thermo Fisher Scientific-CN (Shanghai, China), formic acid for mass spectrometry was purchased from Sigma, St. Louis, MO, USA, 5 × TransScript All-in-one SuperMix and gDNA Remover were purchased from TransGen, Beijing, China, the TRIzon reagent was from CWBIO, Taizhou, Jiangsu, China, the water used for the experiments was ultrapure, and the other reagents used were all analytically pure. The ultrafast centrifuge, ultrasonic crusher, Nanodrop 2000, and vacuum centrifuge concentrator were from Thermo Corporation, Waltham, MA, USA, Sonic Corporation, Stratford, CT, USA, and Genevac Corporation, Ipswich, UK, respectively.

### 2.2. Animals

Six pregnant SPF SD rats (gestational age 15–18 days) were purchased from Vital River Laboratories. Rats were housed in a room with a temperature of 21–24 °C, humidity of 45–55%, and a daily cyclic indoor light/dark cycle of 12 h/12 h (9:20–21:20 lighting). All of the experimental procedures were carried out strictly according to the operational guidelines of the China Astronaut Center, and approved by the Animal Ethics Committee of the Institute of Medicinal Plant Development, Chinese Academy of Medical Sciences.

### 2.3. Animal Experimental Procedures

The delivery of pregnant rats was observed every 12 h before the expected delivery date, and the birth date of the offspring rats was recorded as PND0 and randomly divided into control (CON) and maternal separation (MS) groups. For the MS group, starting from PND2, the mother rats were removed from the home cage and placed in a different cage every day, while the pups were transferred from the home cage to another clean cage with some bedding and nesting material from the home cage to preserve the pups’ smell, which was placed in a different room to avoid communication between the mother and the pups. After separation, the pups and the mother rat were placed back into their home cages successively. MS was performed continuously at 14:00–17:00 for 3 h per day from PND2 to PND8. After the pups were weaned at PND22, the offspring rats of different groups and sexes were kept in separate cages, with 4–6 rats per cage, awaiting the subsequent experimental manipulations.

**Figure 1:**
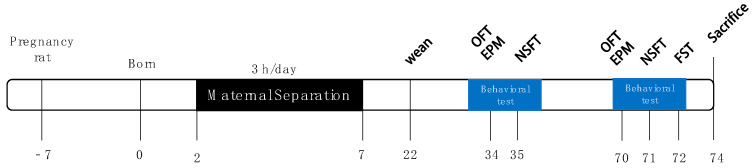
Animal experiment process. OFT: Open Field Test; EPM: Elevated Plus Maze; NSFT: Novelty Suppressed Feeding Test; FST: Forced Swimming Test.

The experimental procedure is shown in Figure 1. 

### 2.4. Behavioral Tests

#### 2.4.1. Open Field Test (OFT)

The OFT was originally proposed by Hall [12] to detect spontaneous activity and exploratory behavior of animals in unfamiliar environments, which can be used to evaluate their voluntary activity and anxiety-like behavior. The rats are placed in the center of an open field (a black circular apparatus with an inner diameter of 80 cm and a height of 50 cm) for 5 min, and the multifunctional detector will record the movement distance, movement time, and the distance and time moved in center area.

#### 2.4.2. Elevated Plus Mazes (EPM)

EPM was used to assess the anxiety behavior of the animals according to the method established by Pellow [13]. The rat EPM apparatus consists of two 50 cm × 10 cm open arms, two 50 cm × 10 cm × 40 cm closed arms, and a 10 cm × 10 cm center area, and the maze is 70 cm above the ground. The rats were put in the center area with their heads facing the open arms, and the test was carried out for 5 min. The computer program continuously detected entries the rats made through the open and closed arms and the times the entries occurred, and calculated the open arm entries (%) and duration (%) during the 5 min.

#### 2.4.3. Novelty Suppressed Feeding Test (NSFT)

Referring to the NSFT methodology established by Britton [14], the rats were fasted for 12 h so that they were in a hungry state, and when they were placed in a novel environment, a conflict between the desire to feed and a fear of the new environment arose. Food which was small enough to be picked up and eaten by rats was put in the center of a black square field with a size of 76 cm × 76 cm × 46 cm, and the rats were placed at the edge of the square field. The time taken by the rats to explore the environment until they picked up the food and ingested it was the latency to feeding, and the latency to feeding was monitored over a period of 15 min.

#### 2.4.4. Forced Swimming Test (FST)

The FST is commonly used to detect depression-like behavior. The method referred to Slattery et al. [15], the rats were placed in a cylindrical transparent swimming apparatus with an inner diameter of 20 cm and a height of 50 cm, and the water depth was adjusted to be 30 cm, and the water temperature was adjusted to be 25 ± 1 °C. Then, 24 h prior to the formal test, each rat underwent a 15 min swimming session. The immobility time of the rats for 5 min was counted by video recording with a DV camera. The immobility time was determined by the rats floating on the water surface, only making small movements to maintain body balance and exposing their heads to the water surface. The water was changed after each rat was tested. Attention was paid to keeping the test environment quiet and avoiding disturbing the rats.

### 2.5. Collection of Biological Samples

Then, 12 h after behavioral test, the rats were anesthetized by an intraperitoneal injection of 20% urethane saline solution, and the hippocampus was isolated from the brain, rapidly plunged into liquid nitrogen and subsequently transferred to a −80 °C refrigerator for storage.

The whole blood was collected in centrifuge tubes from the abdominal aorta, placed in a refrigerator at 4 °C for stratification, then the supernatant was extracted, followed by centrifugation at 3500 rpm/min for 15 min at 4 °C. The supernatant was aspirated and put into centrifuge tubes, and then transferred to a refrigerator at −80 °C for storage.

### 2.6. Determination of Serum Corticosterone and Hippocampal NAD+ and NADH

Serum corticosterone content was measured by Corticosterone Assay Kit (H205-1-2) from Nanjing Jiancheng Bioengineering Institute, Nanjing, China. Hippocampal NAD and NADH content were measured by a WST-8 Enhanced NAD+/NADH Assay Kit from Beyotime Biotech Incorporated, Shanghai, China.

### 2.7. Determination of Hippocampal Inflammatory Cytokines by Real-Time Fluorescence Quantification (qRT-PCR)

The total RNA preparation of the collected hippocampal samples was similar to our published study [16]. Briefly, the total RNA was extracted by TRIzon reagent and the concentration was determined by Nanodrop 2000. The mixture (5 μL RNA (200 ng/μL), 4 μL 5 × TransScript All-in-one SuperMix, 1 μL gDNA Remover and 10 μL RNase-free water) was reacted under a specific condition to obtain the cDNA. Finally, the same amount of cDNA was applied in a Bio-Rad CFX96 Real-Time PCR Detection System for qRT-PCR analysis. A three-step amplification program was used: 94 °C for 30 s followed by 45 cycles of amplification (94 °C for 5 s, 60 °C for 15 s, and 72 °C for 10 s) and finally a melting curve program.

### 2.8. Hippocampal Untargeted Metabolomics Using UPLC-Q-TOF/MS

#### 2.8.1. Hippocampal Tissue Processing

Hippocampal tissue was removed from a −80 °C refrigerator, thawed on ice, accurately weighed to 50 mg, placed in a 2 mL centrifuge tube, 500 μL ultrapure water was added, homogenized for 2 min, then 1000 μL acetonitrile was added to precipitate proteins, vortexed for 30 s, and centrifuged at 13,000 rpm for 15 min. The supernatant was taken, blown dry with nitrogen, and redissolved with 100 µL acetonitrile–water (10:90, *v*/*v*), centrifuged at 13,000 rpm for 15 min, filtered with 0.22 µm membrane. The sample was kept in the sample chamber at 4 °C for 1 h, and then analyzed.

#### 2.8.2. UPLC-Q-TOF/MS Analysis

Liquid chromatographic conditions: Waters ACQUITY UPLC system (Waters Corp., Milford, MA, USA) with a binary solvent delivery system and an autosampler module was used. The chromatographic column was a HSS T3 column (100 mm × 2.1 mm, 1.8 μm, Waters Corp., Milford, MA, USA); mobile phase A: acetonitrile (containing 0.1% formic acid), mobile phase B: water (containing 0.1% formic acid); the column temperature was maintained at 40 °C; the temperature of the sample compartment was maintained at 4 °C; the flow rate was 0.45 mL/min; the sample was injected with 5 μL at a time; the mobile phase linear gradient procedure for all samples was as follows: 0–0.5 min, 1% B; 0.5–4 min, 1–60% B; 4–10 min, 60–99% B; 10–13 min, wash with 100% B; 13–15 min, equilibrate with 1% B.

Mass spectrometry conditions: mass spectrometry data were collected using a SYNAPT G2 HDMS Q-TOF mass spectrometer (Waters Corp., Manchester, UK) equipped with an electrosprayionization (ESI) that operates in both positive and negative ion scanning modes. Optimized parameters for the TOF MS are given in [17]. Continuous medium mode data were collected, and the mass range was set from mass-to-charge ratios (*m*/*z*) 50 to 1200.

#### 2.8.3. Data Processing

Firstly, the mass spectrometry data (.raw) were converted to mzXML format using the ‘msconvert’ function in ProteoWizard (Version 3.0), followed by data processing and identification of characteristic peaks using the R (Version 4.3.1)-based xcms package, which provides a table containing retention times, *m*/*z*, and peak areas for each metabolite for each sample.

#### 2.8.4. Multivariate Analysis of UPLC-Q-TOF/MS Data

Principal component analysis (PCA) and orthogonal partial least squares-discriminant analysis (OPLS-DA) were performed by SIMCA-P software (Umetrics AB, Umeå, Sweden, version 14.1) to identify patterns in the data, and to examine the separation profiles of data between the CON and MS groups.

The explanatory ability (expressed in R2) and predictive ability (expressed in Q2) of the established PCA model were evaluated, and the predictive ability of the OPLS-DA model was assessed using the permutation test. Variables that distinguish the difference between the two groups were found from the established OPLS-DA model by variable important in the projection (VIP). Difference variables were analyzed for significance using two-sided *t*-test and *p* < 0.05 was statistically significant. Finally, differential metabolic pathways were analyzed by KEGG (Kyoto Encyclopedia of Genes and Genomes) pathway enrichment analysis using MetaboAnalyst 6.0 (https://www.metaboanalyst.ca/, accessed on 8 August 2024).

### 2.9. Statistical Analysis

The data analysis was performed using SPSS 25.0 software. Normality test was performed using Shapiro–Wilk test, and parametric test method was used for data that conformed to normal distribution. Statistical methods for body weight were performed using two-way ANOVA to assess the effects of stress and age, and their interaction in the three weeks after birth, multi-way ANOVA to assess the effects of stress, sex and age, and their interaction after three weeks of age, and two-way ANOVA to assess the effects of stress and sex, and their interaction for the other data, and post hoc tests were performed using Least Significant Difference (LSD) test. Data with heterogeneous variance were analyzed by Dunnett’s *t* test. Data that did not conform to the normal distribution were analyzed using a nonparametric test, and Mann–Whitney test was used to analyze differences in means. Pearson’s analysis was used to explore correlations between behavioral indicators and metabolite changes. GraphPad 8.0 was used for graphing, and experimental data were expressed as mean ± standard error (Mean ± SEM), with *p* < 0.05 indicating statistical significance.

## 3. Results

### 3.1. Effect of One-Week MS on Body Weight of Offspring Rats

**Figure 2:**
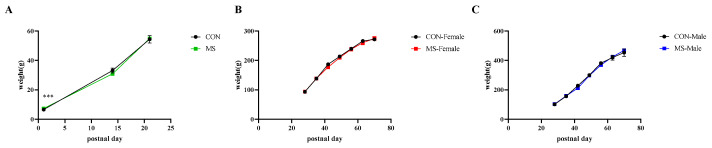
Curve plot of body weight changes in one-week MS rats. The data represented mean ± SEM, CON: control group, MS: maternal separation group; compared with control group, *** *p* < 0.001; (**A**), curve plot of body weight change in all rats (*n* = 20); (**B**), curve plot of body weight change in female rats (*n* = 8–12); (**C**), curve plot of body weight change in male rats (*n* = 8–12).

As it was unable to accurately distinguish the sex of the pups within three weeks of birth, we monitored the body weights of the entire litter. After weaning and grouping by sex, the body weight was recorded separately by sex once a week. In the three weeks after birth, only the age main effect had a significant effect on body weight (F (2, 119) = 729.786, *p* < 0.001), after three weeks, the interaction effect of age and sex had a significant effect on body weight (F (6, 279) = 115.221, *p* < 0.001). As shown in Figure 2, the MS had no significant effect on the body weight of both male and female rats, except for PND1.

Since the grouping was based on litters rather than individual pups, the obvious weight differences in PND1 were due to differences in pups at birth rather than MS stress.

### 3.2. Effect of One-Week MS on Adolescent Behaviors of Offspring Rats

**Figure 3:**
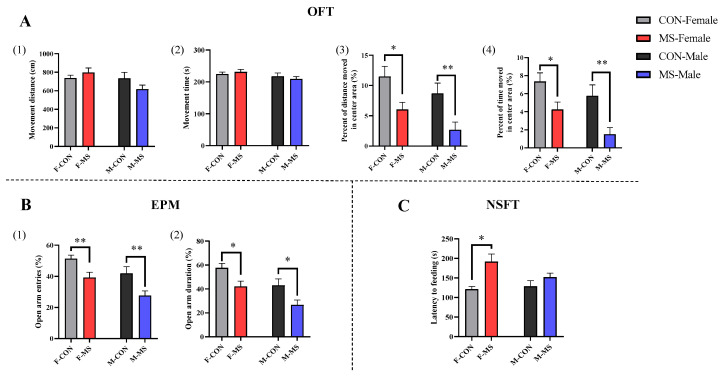
Effect of one-week MS on adolescent behavior of offspring rats. The data represented mean ± SEM, *n* = 8–12. CON: control group, MS: maternal separation group; compared with control group, * *p* < 0.05, ** *p* < 0.01; (**A**), OFT: open field test, (1) movement distance (2) movement time (3) percent of distance moved in center area (%) (4) percent of time moved in center area (%), (**B**), EPM: elevated plus mazes, (1) open arm entries (%) (2) open arm duration (%), (**C**), NSFT: novelty suppressed feeding test.

Considering that the FST is more stimulating to animals, the relatively less stimulating tests of OPT, EPM and NSFT were performed during adolescence. In the OFT, there were no effects of the main and interaction effects of sex and stress on either movement distance or movement time (Figure 3A(1,2), *p* > 0.05). There were significant effects of sex main effect (F (1, 33) = 4.290, *p* = 0.047) and stress main effect (F (1, 33) = 15.009, *p* = 0.001) on the percent of movement distance in the center area, and there were significant effects of sex main effect (F (1, 34) = 5.314, *p* = 0.028) and stress main effect (F (1, 34) = 15.452, *p* < 0.001) on the percentage of movement time in the center area. As compared to the CON group, there were significant decreases in the percent of movement distance and movement time in center area (Figure 3A(3), *p* < 0.05 for both females and males; Figure 3A(4), *p* < 0.05 for females and *p* < 0.01 for males). In the EPM, there were significant effects of sex main effect (F (1, 37) = 10.911, *p* = 0.002) and stress main effect (F (1, 37) = 17.252, *p* < 0.001) on the open arm entries; significant effects of sex main effect (F (1, 37) = 11.996, *p* = 0.001) and stress main effect (F (1, 37) = 13.689, *p* = 0.001) on the open arm duration. The open arm entries and the open arm duration were significantly decreased in both female and male MS offspring rats (Figure 3B(1), *p* < 0.01 for both females and males; Figure 3B(2), *p* < 0.05 for both females and males). In the NSFT, there was only significant effect of sex main effect (F (1, 36) = 11.696, *p* = 0.002) on the latency to feeding. It was significantly prolonged in MS offspring female rats (Figure 3C, *p* < 0.01), whereas there was only a tendency in MS offspring male rats. These results suggested that one-week MS induced anxiety-like behavior in adolescent offspring in both male and female rats.

### 3.3. Effect of One-Week MS on Adulthood Behaviors of Offspring Rats

**Figure 4:**
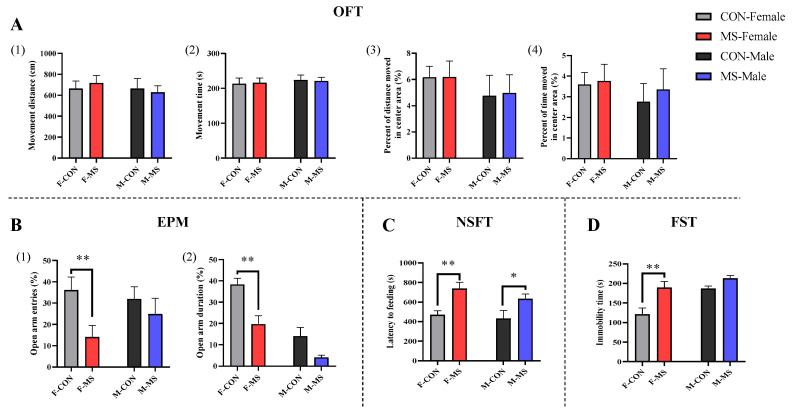
Effect of one-week MS on adulthood behavior of offspring rats. The data represented mean ± SEM, *n* = 8–12. CON: control group, MS: maternal separation group; compared with control group, * *p* < 0.05, ** *p* < 0.01; (**A**), OFT: open field test, (1) movement distance (2) movement time (3) percent of distance moved in center area (%) (4) percent of time moved in center area (%), (**B**), EPM: elevated plus mazes, (1) open arm entries (%) (2) open arm duration (%), (**C**), NSFT: novelty suppressed feeding test, (**D**), FST: forced swimming test.

In addition to detecting anxiety-like behaviors, the FST was added to detect depression-like behavior during adulthood. In the OFT, there were no significant effects of sex, stress main effects, or interaction effects on any of the four indicators. In the EPM, there was only a significant effect of stress main effect (F (1, 39) = 6.632, *p* = 0.014) on the open arm entries; and there were significant effects of sex main effect (F (1, 39) = 9.543, *p* = 0.004) and stress main effect (F (1, 39) = 9.543, *p* = 0.004) on the open arm duration. Compared with the CON group, the open arm entries and open arm duration were significantly decreased in MS female rats, and only tended to decrease in MS male rats (Figure 4B(1,2), *p* < 0.01 for females and *p* < 0.05 for males). There was only significant effect of stress main effect (F (1, 36) = 14.867, *p* = 0.001) on the latency to feeding in NSFT, and there were significant effects of sex main effect (F (1, 37) = 10.613, *p* = 0.003) and stress main effect (F (1, 37) = 11.578, *p* = 0.002) on the immobility time in FST. The latency to feeding was significantly prolonged in both MS female and male rats (Figure 4C, *p* < 0.01 for females and *p* < 0.05 for males). The immobility time, however, was significantly prolonged only in female rats (Figure 4D, *p* < 0.01 for females). Summarily, MS developed anxiety- and depression-like behaviors during adulthood in both female and male offspring rats.

### 3.4. Effect of One-Week MS on Serum Corticosterone (CORT) of Offspring Rats

**Figure 5:**
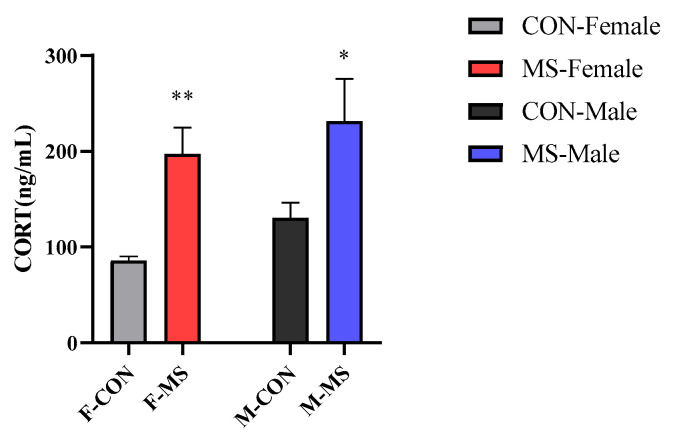
Effect of one-week MS on serum CORT of offspring rats. The data represented mean ± SEM, *n* = 6–8. CON: control group, MS: maternal separation group; Compared with control group, * *p* < 0.05, ** *p* < 0.01.

Only the main effect of stress (F (1, 28) = 18.015, *p* < 0.001) caused significant effects on serum corticosterone levels. Compared with CON group, serum CORT levels were significantly increased in both female and male MS rats (Figure 5, *p* < 0.01 for females and *p* < 0.05 for males).

### 3.5. Effect of One-Week MS on Hippocampal Inflammatory Cytokines of Offspring Rats

**Figure 6:**
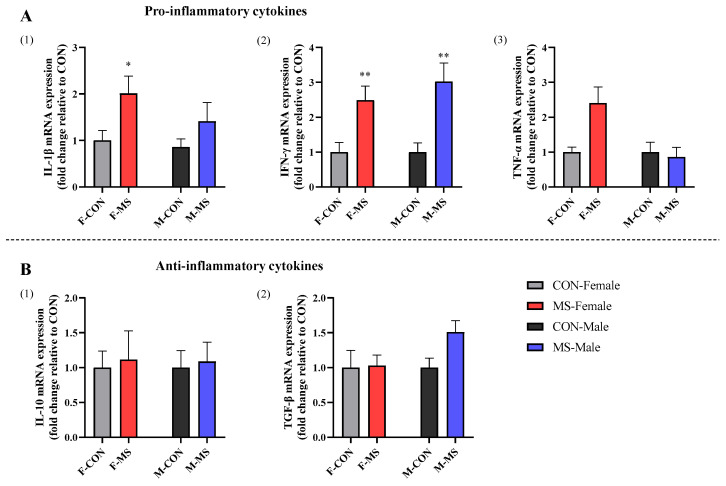
Effect of one-week MS on hippocampal inflammatory cytokines of offspring rats. The data represented mean ± SEM, *n* = 6–8. CON: control group, MS: maternal separation group; compared with control group, * *p* < 0.05, ** *p* < 0.01. (**A**), Effect of MS on pro-inflammatory cytokine expression in offspring rats, (1) IL-1β mRNA expression (2) IFN-γ mRNA expression (3) TNF-α mRNA expression, (**B**), Effect of MS on anti-inflammatory cytokine expression in offspring rats, (1) IL-10 mRNA expression (2) TGF-β mRNA expression.

Only the stress main effect had significant effects on interleukin-1β (IL-1β) (F (1, 30) = 6.727, *p* = 0.015) and interferon-gamma (IFN-γ) (F (1, 27) = 23.431, *p* < 0.001) mRNA expression. Interaction effects of sex and stress significantly affect the mRNA expression of tumor necrosis factor (TNF-α) (F (1, 29) = 6.478, *p* = 0.017). As shown in Figure 6A, the pro-inflammatory cytokines of IFN-γ and IL-1β were significantly elevated in MS female rats (*p* < 0.05 and *p* < 0.01), and only IFN-γ were significantly elevated in MS male rats (*p* < 0.01).

The main and interaction effects of sex and stress were not significant for the anti-inflammatory factor interleukin-10 (IL-10) and transforming growth factor-β (TGF-β) (Figure 6B, *p* > 0.05).

### 3.6. Effect of One-Week MS on Hippocampal Untargeted Metabolomics of Offspring Rats

#### 3.6.1. Validation of Analytical Methods

The analysis method referred to the published literature [18]. In order to ensure the reliability of the established UPLC-Q-TOF/MS method for hippocampal sample analysis, QC samples were applied to evaluate the precision of the method and the stability of the instrument. Before the formal analysis, QC samples were collected on10 consecutive occassions to evaluate the precision of the analysis method. During sample analysis, every 10 samples were analyzed, accompanied by one injection of QC sample to evaluate the stability of the instrument.

Ten characteristic peaks with different retention times and *m*/*z* were selected. The results showed that the RSD of peak area varied from 4.79% to 20.51% in positive ion mode, and from 5.83% to 24.66% in negative ion mode, respectively (Table 1), indicating that the precision of the method and the stability of the instrument reached the requirements of analytical methods, and could be used for metabolomics data collection of hippocampal samples.

#### 3.6.2. Analysis of Metabolic Profile in the Hippocampus

**Figure 7:**
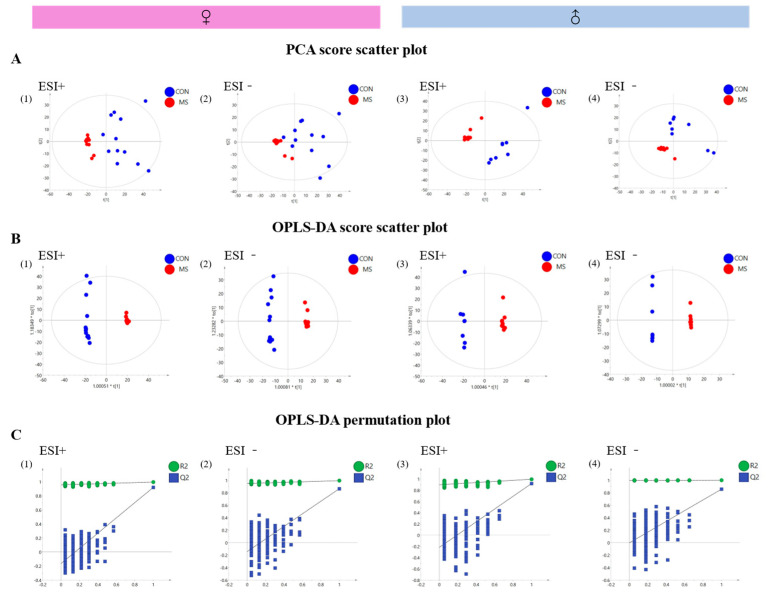
Untargeted metabolomic profile analysis of hippocampus in MS female and male rats. The data represented mean ± SEM, *n* = 8–12, (**A**), Scatterplot of PCA, CON (control group, blue dots); MS (maternal separation group, red dots) (1) PCA scatterplot of positive ion metabolomic profiles in female rats (R2Y = 0.996, Q2 = 0.92), (2) PCA scatterplot of negative ion metabolomic profiles in females (R2Y = 0.994, Q2 = 0.864), (3) PCA scatterplot of positive ion metabolomic profiles in male rats (R2Y = 0.996, Q2 = 0.918), (4) PCA scatterplot of negative ion metabolomic profiles in male rats (R2Y = 1, Q2 = 0.856); (**B**), OPLS-DA analysis scatterplot, CON (blue dots); MS (red dots), (1) female rat positive mode OPLS-DA scatterplot (R2Y = 0.996, Q2 (cum) = 0.92), (2) female rat negative mode OPLS-DA scatterplot (R2Y = 0.994, Q2 (cum) = 0.864), (3) male rat positive mode OPLS-DA scatterplot (R2Y = 0.996, Q2 (cum) = 0.918), (4) male rat negative mode OPLS-DA scatterplot (R2Y = 1, Q2 (cum) = 0.856); (**C**), OPLS-DA permutation test plot, R2 (green dots) Q2 (blue squares), (1) female rat positive OPLS-DA permutation test plot (R2Y = 0.957, Q2 = −0.143), (2) female rat negative OPLS-DA permutation test plot (R2Y = 0.948, Q2 = −0.142), (3) male rat positive OPLS-DA permutation test plot (R2Y = 0.891, Q2 = −0.229), and (4) male rat negative OPLS-DA permutation test plot (R2Y = 0.997, Q2 = −0.00434).

Firstly, the PCA method was used to examine the separation of data contours between the CON and MS groups. Both MS female and male rats showed significant separation from CON rats in positive and negative ion modes (Figure 7A(1–4)), indicating that MS stress caused significant changes in the hippocampal metabolism of offspring rats.

Then, the OPLS-DA method was used to further analyze the profiles of the two groups, which showed that both MS female and male rats were completely separated from CON rats under positive and negative ion modes (Figure 7B(1–4)).

To examine whether there was any overfitting of the supervised OPLS-DA model, the permutation test was performed. And it can be seen that all four models have a good prediction (Q2 < 0, Figure 7C(1–4)), indicating that the model was reliable. 

#### 3.6.3. Identification of Differential Metabolites in the Hippocampus

**Figure 8:**
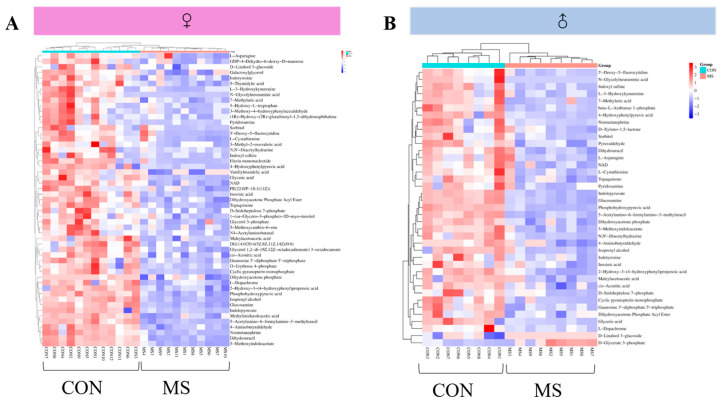
Heat map of differential metabolite changes in male and female rats. (**A**), Heat map of differential metabolite changes in female rats; (**B**), Heat map of differential metabolite changes in male rats.

This study focused on the metabolites with variable importance in the projection greater than 1 (VIP > 1) and significant difference in *t*-test (*p* < 0.05). By comparing human metabolome database (HMDB) and manually checking tandem mass spectrometry fragments, a total of 57 differential metabolites were identified, of which 53 were identified in female MS rats and 39 in male MS rats. Their variations are presented in the heat map in Figure 8. There were 35 differential metabolites observed in both MS female and male rats, and all of which showed a significant decrease, except for a significant increase in D-Linalool 3-glucoside in male rats. In addition, there were 18 differential metabolites observed only in female rats, and only 4 in male rats (Table 2).

#### 3.6.4. Metabolic Pathway Analysis on the Differential Metabolites in the Hippocampus

**Figure 9:**
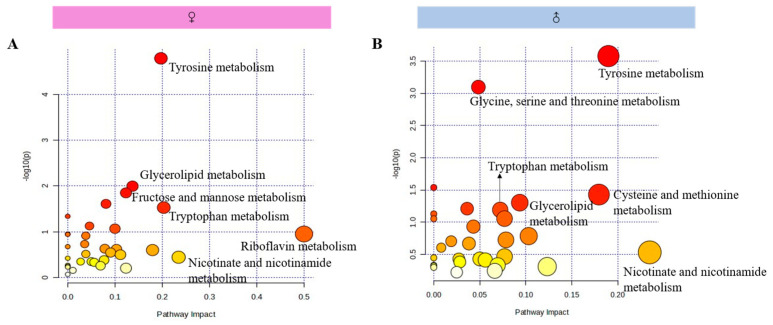
Pathway analysis of differential metabolites of hippocampus in female and male rats. The redder the color, the larger the -LOG(p) value, and the larger the bubble, the larger the Pathway Impact value. (**A**), Pathway analysis of differential metabolites of hippocampus in female rats; (**B**), Pathway analysis of differential metabolites of hippocampus in male rats.

The metabolic pathways were analyzed by KEGG pathway enrichment analysis performed on the differential metabolites (Table 2), the number of differential metabolites found to be involved in metabolic pathways were shown in Table 3, and metabolic pathway enrichment was analyzed as in Figure 9.

#### 3.6.5. Correlation Analysis of Behavior Data and Hippocampal Metabolites

**Figure 10:**
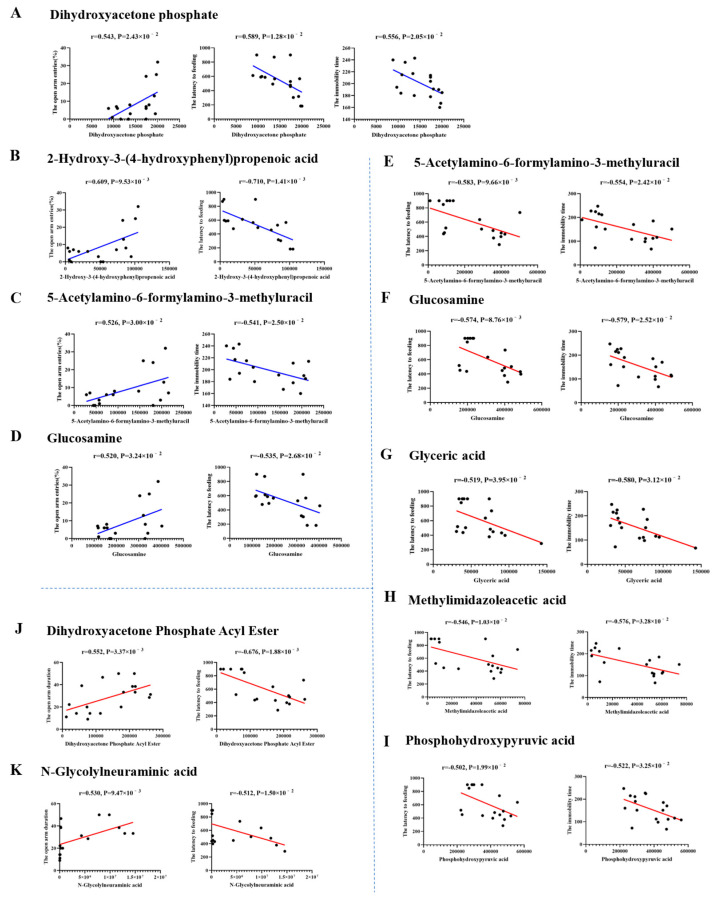
The main correlation of behavior and hippocampal differential metabolites in MS female and male rats. The horizontal coordinate is the behavior data, the vertical coordinate is the metabolite intensity, the blue line shows the correlation in males and the red line shows females.

In order to explore the relationship between behavior and hippocampal differential metabolites in MS rats, the behavioral data of the EPM, NSFT, and FST in adulthood and hippocampal differential metabolites were subjected to Pearson correlation analysis. The r > 0.5 and *p* < 0.05 were considered as significant correlation. The results showed that 27 differential metabolites in female rats and 13 in male rats were significantly correlated with behavior data, respectively. Among these correlated metabolites, only Dihydroxyacetone phosphate was significantly correlated with three tested behaviors, and 2-hydroxy-3-(4-hydroxyphenyl)propenoic acid, 5-acetylamino-6-formylamino-3-methyluracil, and glucosamine were significantly correlated with NSFT and FST data in male rats. Meanwhile, five metabolites of 5-acetylamino-6-formylamino-3-methyluracil, glucosamine, glyceric acid, methylimidazoleacetic acid, and phosphohydroxypyruvic acid in MS female rats were significantly correlated with NSFT and FST data, and dihydroxyacetone phosphate acyl ester and N-glycolylneuraminic acid showed an obvious correlation with EPM and NSFT data. The main correlation analysis scatter plots are shown in Figure 10.

### 3.7. Effect of One-Week MS on Hippocampal NAD+ and NADH of Offspring Rats

**Figure 11:**
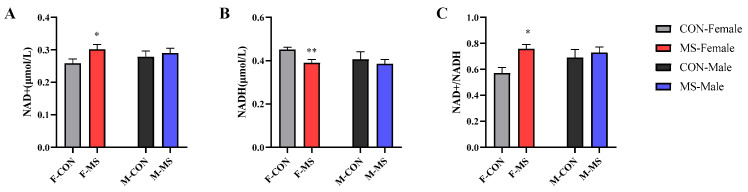
Effect of one-week MS on hippocampal NAD+ and NADH of offspring rats. The data represented mean ± SEM, *n* = 6–8. CON: control group, MS: maternal separation group; compared with control group, * *p* < 0.05, ** *p* < 0.01. (**A**), NAD+; (**B**), NADH; (**C**), ratio of NAD+/NADH.

The main and interaction effects of sex and stress were not significant for both NAD+ and NADH. While the main effect of stress had a significant effect on the ratio of NAD+/NADH (F (1, 31) = 5.974, *p* = 0.021). As shown in Figure 11, as compared to the CON group, NAD+ was significantly elevated (*p* < 0.05), NADH was significantly reduced (*p* < 0.01), and the ratio of NAD+/NADH was significantly elevated (*p* < 0.05) in female rats, with no significant difference in male rats.

## 4. Discussion

### 4.1. One-Week MS Induced Sex-Specific Anxiety- and Depression-like Behaviors in Offspring Rats During Adolescence and Adulthood

As a classic early stress factor, MS can simulate the effect of maternal loss on the physiological and psychological development of offspring in the early development of human beings [19,20,21]. In the present study, the offspring rats were separated from their mothers for one week from PND2 to 8, the behavioral detection showed that both MS female and male rats showed anxiety- and depression-like behaviors during adolescence and adulthood, which were similar to the reported literature on two- or three weeks MS research [22,23,24,25]. Comparing the serious effects on the anxiety-like behavior of the behavior results, MS female rats significantly prolonged the latency to feeding in NSFT during adolescence and significantly decreased the open arm entries and duration in EPM, and significantly prolonged the immobility time in FST during adulthood, whereas MS male rats showed no significant differences in these tests, suggesting that MS for the first week after birth has more effect on female rats.

### 4.2. One-Week MS Induced Sex-Specific Effects of HPA Axis Overactivation and Hippocampal Inflammation in Offspring Rats

A large number of studies have shown that anxiety and depression are related to the overactivation of hypothalamic–pituitary–adrenal (HPA) axis, which is very important for the maintenance of internal environmental homeostasis in most organisms. Although there is a stress hyporesponsive period in the neonatal period of human and rodents [26], which protects the normal development of the HPA axis, but when exposed to intense stimulation, the HPA axis development will be affected, causing physiological and behavioral changes in adulthood [27,28]. Under normal circumstances, the HPA axis is regulated by negative feedback to maintain the normal concentrations of corticosterone, an end effector of HPA axis, which plays an important role in stress response. In the present study, the results of serum corticosterone of MS rats were significantly elevated in both females and males as compared to the control group (Figure 5), suggesting that one-week of MS stress affected the normal development of HPA axis, resulting in an overactivation of the HPA axis in the whole growth period.

Additionally, it has demonstrated that an overactivation of the HPA axis and perennial high levels of corticosterone impaired metabolism as well as produced chronic inflammation [29,30,31]. Our data displayed that the pro-inflammatory factors of IFN-γ and IL-1β were significantly elevated in MS female rats; meanwhile, only IFN-γ increased in MS male rats, indicating that MS had a stronger pro-inflammatory response to the hippocampus of female rats than to that of male rats.

### 4.3. One-Week MS Showed Sex-Specific Effects on Hippocampal Metabolomics in Adulthood Offspring Rats

Based on the behavioral experiments and hippocampal inflammatory results, it was hypothesized that the metabolism of hippocampus cells must have changed. Untargeted metabolomics can comprehensively reflect the overall profile of cellular metabolism; therefore, the UPLC-Q-TOF/MS technique was used to study the hippocampal untargeted metabolomics of MS rats in this study. The identification of differential metabolites in hippocampus indicated that there were 35 metabolites observed in both MS female and male rats, and sex-specific differential metabolites showed 18 metabolites observed only in female rats, and 4 only in male rats (Table 2). The pathway analysis of differential metabolites showed that 28 common metabolic pathways were involved in female and male rats, and sex-specific metabolic pathways included 5 in female rats and 2 in male rats. Among the common metabolic pathways, the sequence of pathway changes in female and male rats were different. From the above data, it indicated that one-week MS had sex-differentiated characteristics on the hippocampal metabolism of offspring rats. From the types of metabolites and the number of metabolic pathways, it can be seen that the effect of one-week MS on the hippocampus metabolism of female offspring rats is greater than that of male rats. Due to the difference in animal strains, tissue sites and treatments, and analytical methods used to conduct the untargeted metabolomics studies, the identified metabolites were different. However, in terms of metabolic pathway, our results showed similarities with other studies [32,33].

### 4.4. One-Week MS Induced Energy Metabolism Disorder in Hippocampal Cells of Offspring Rats

Due to the specific functions of neurons, a very high energy consumption of neurons is required. In general, neurons produce ATP primarily through mitochondrial oxidative phosphorylation (OXPHOS). As the brain has no glycogen reserves, glucose is almost the sole energy source. The glucose in the brain includes the transport from the periphery via blood circulation, as well as intracerebral gluconeogenesis. The aerobic oxidation of glucose includes three main stages: glycolysis, tricarboxylic acid (TCA) cycle and OXPHOS. Glycolysis provides a series of intermediates that offer precursors for the TCA cycle. Therefore, any substance that can be converted to glucose or enter the TCA cycle is called a gluconeogenic substance. As shown in Table 2, eight possible gluconeogenic substances (galactosylglycerol, sorbitol, L-asparagine, glyceric acid, L-cystathionine, pyruvaldehyde, D-erythrose 4-phosphate and D-sedoheptulose 7-phosphate) in both MS female and male rats were obviously decreased; and another seven possible gluconeogenic metabolites (phosphohydroxypyruvic acid, D-linalool 3-glucoside, glycerol 1,2-di-(9Z,12Z-octadecadienoate) 3-octadecanoate, glycerol 3-phosphate, DG(14:0/20:4(5Z,8Z,11Z,14Z)/0:0), dihydroxyacetone phosphate and D-glycerate 3-phosphate) were significantly reduced only in females, cis-aconitic acid was significantly reduced only in males, indicating that the decrease in potential energy substance in hippocampus of MS female rats is much more than that of male rats.

OXPHOS is the coupling process by which energy is released during the oxidation of a substance in the body and used to phosphorylate ADP to synthesize ATP via the electron transport chain, also called the respiratory chain. The electron transport chain is a system consisting of a series of electron carriers that transfer electrons from NADH and FADH2 to oxygen. And flavin mononucleotide (FMN) participates in respiratory chain composition as a cofactor of NADH dehydrogenase, which can also bind a molecule of adenylate (AMP) to generate FAD, which is directly involved in hydrogen and electron transfer. From the untargeted metabolomics, FMN showed reduction only in female rats.

As NAD (including NAD+ and NADH) plays a key role in regulating a variety of physiological processes such as energy metabolism, which NADH is utilized by the electron transport chain in mitochondria and is involved in oxidative phosphorylation to produce ATP, we tested the contents of NAD+ and NADH using an assay kit. The data showed a significant increase in NAD+, a significant decrease in NADH, in MS female rats, and no obvious change in MS male rats (Figure 11). From the gluconeogenic substance, the electron transport chain of OXPHOS as well as NAD contents, the energy metabolism disorder in hippocampus of MS female rats was more serious than that of MS male rats.

Additionally, published studies showed that impaired energy metabolism is the main characteristic of depression and anxiety in animals [34,35,36] and the reduction in NADH, FMN, dihydroxyacetone phosphate, and cis-aconitic acid were observed in depressed animals [37,38,39], which further proved that MS-induced anxiety- and depression-like behaviors in the offspring rats may be related to the impaired energy metabolism in the hippocampus.

### 4.5. One-Week MS Induced Disfunction of Hippocampal Tyrosine and Tryptophan Metabolism in Offspring Rats

Tyrosine is an important amino acid, which can be converted into a variety of biologically active substances, such as adrenaline, norepinephrine, dopamine, tetrahydrobiopterin, uranyluronic acid, and melanin through different metabolic pathways, and plays an important physiological function in the human body. As an essential amino acid, tryptophan absorbed from food is mainly metabolized through three pathways, namely 5-hydroxy-trytamine (5-HT), kynurenine and indole pathways, to produce a series of active substances such as 5-HT, kynurenine, 3-hydroxykynurenine, quinolinic acid, kynurenine quinolinic acid, and NAD+. Previous studies have shown that metabolism of tyrosine and tryptophan plays a central role in the pathogenesis of many neurological and psychiatric disorders [40]. As shown in Table 3, MS caused eight tyrosine and four tryptophan metabolites—a significant reduction in hippocampus of the offspring rats. In particular, NE and 5-hydroxy-L-tryptophan (5-HTP), the precursor of 5-HT, showed a significant decrease, and the decrease in neurotransmitter of NE and 5-HT are the important features of depression and anxiety [41].

In summary, from the above data, it can be seen that MS in the first week after birth can induce anxiety- and depression-like behavior and hippocampal metabolic disorders in male and female offspring rats, mainly because one-week MS over-activated the HPA axis and increased the serum content of its end effector, corticosterone. As the hippocampus is a brain region with a high expression of corticosterone receptors [42], the metabolic disorders in hippocampus may be related to the increase in corticosterone content. Meanwhile, we also found that one-week MS had a greater effect on the behavior and hippocampal metabolism of female offspring rats than of male rats, which may be related to the similar but not identical brains of male and female rats, and growing evidence shows sex differences at finer levels, such as differences in synaptic patterns [43,44] and neuron densities [45,46,47].

## 5. Conclusions

One-week MS after birth induced sex-specific anxiety- and depression-like behaviors in female and male offspring rats during adolescence and adulthood, as well as sex-differentiated characteristics in the hippocampus metabolomics of adult MS rats. The hippocampus metabolomics showed inflammatory factor changes, the identification of differential metabolites, metabolic pathways, and energy metabolism disfunction in MS female rats that were different from MS male rats. From the experimental data, the effects of MS on the offspring female rats were more severe than those on the male rats, and anxiety- and depression-like behaviors induced by MS may be related to hippocampal energy disorder and neurotransmitter imbalance.

## Figures and Tables

**Table 1:** Evaluation of precision and stability of UPLC-Q-TOF/MS analyses.

ESI+					ESI−				
No.	RT (min)	*m*/*z*	Precision RSD (×100%)	Stability RSD (×100%)	No.	RT (min)	*m*/*z*	Precision RSD (×100%)	Stability RSD (×100%)
1	1.63	204.6211	0.0479	0.0802	1	0.49	130.9669	0.0575	0.2138
2	1.75	306.0644	0.0215	0.1830	2	0.49	174.9561	0.0339	0.2112
3	1.95	663.3329	0.0347	0.1778	3	1.36	303.0507	0.0528	0.2080
4	2.13	998.5831	0.0364	0.1853	4	1.45	467.0912	0.0321	0.1786
5	2.24	646.0117	0.0198	0.0687	5	1.76	358.5540	0.0535	0.1677
6	2.24	861.7346	0.0305	0.0735	6	1.88	548.1155	0.0348	0.1774
7	2.26	647.0518	0.0401	0.0888	7	2.61	165.0556	0.0432	0.1127
8	2.72	705.2183	0.0396	0.2051	8	3.09	125.0979	0.0484	0.1951
9	4.55	250.1779	0.0186	0.1901	9	6.25	566.3448	0.0583	0.2466
10	8.01	517.3692	0.0369	0.1313	10	6.45	506.3250	0.0565	0.2150

**Table 2:** UPLC-Q-TOF/MS based differential metabolites in MS hippocampus.

Metabolites	RT (min)	*m*/*z*	Adduction	Female CON vs. MS	Male CON vs. MS
d-Glycerate 3-phosphate	3.40	204.0872	[M + NH_4_]^+^	ns	↑ **
d-Xylono-1,5-lactone	17.95	171.1002	[M + Na]^+^	ns	↓ *
Pyruvaldehyde	18.01	95.05016	[M + Na]^+^	ns	↓ **
beta-l-Arabinose 1-phosphate	0.97	231.1141	[M + H]^+^	ns	↓ ***
5-Hydroxy-l-tryptophan	9.50	243.211	[M + Na]^+^	↓ *	ns
d-Erythrose 4-phosphate	1.98	201.0878	[M + H]^+^	↓ **	ns
1-(sn-Glycero-3-phospho)-1d-myo-inositol	4.10	333.206	[M − H]^−^	↓ **	ns
GDP-4-Dehydro-6-deoxy-d-mannose	5.69	588.3307	[M + H]^+^	↓ **	ns
Vanillylmandelic acid	6.35	221.1562	[M + Na]^+^	↓ **	ns
5-Methoxycanthin-6-one	6.42	271.2286	[M + Na − 2H]^−^	↓ **	ns
3-Methoxy-4-hydroxyphenylacetaldehyde	9.04	189.1632	[M + Na]^+^	↓ **	ns
N4-Acetylaminobutanal	11.18	152.1497	[M + Na]^+^	↓ **	ns
(1R)-Hydroxy-(2R)-glutathionyl-1,2-dihydronaphthalene	12.32	490.459	[M + K]^+^	↓ **	ns
PE(22:0/P-18:1(11Z))	2.04	787.1695	[M + H]^+^	↓ ***	ns
DG(14:0/20:4(5Z,8Z,11Z,14Z)/0:0)	2.26	627.8584	[M + K]^+^	↓ ***	ns
Glycerol 1,2-di-(9Z,12Z-octadecadienoate) 3-octadecanoate	2.31	884.4276	[M + H]^+^	↓ ***	ns
Glycerol 3-phosphate	2.87	171.0679	[M − H]^−^	↓ ***	ns
Methylimidazoleacetic acid	5.19	179.1077	[M + K]+	↓ ***	ns
Galactosylglycerol	6.55	255.2431	[M + H]^+^	↓ ***	ns
3-Methyl-2-oxovaleric acid	8.84	169.1011	[M + K]^+^	↓ ***	ns
5-Thymidylic acid	17.95	361.172	[M + K]^+^	↓ ***	ns
Flavin mononucleotide	17.95	479.3335	[M + Na]^+^	↓ ***	ns
d-Linalool 3-glucoside	3.90	339.3827	[M + Na]^+^	↓ *	↑ *
l-Asparagine	4.56	155.1065	[M + Na]^+^	↓ *	↓ *
d-Sedoheptulose 7-phosphate	5.94	291.1621	[M + H]^+^	↓ **	↓ *
Pyridoxamine	9.06	191.1793	[M + Na]^+^	↓ **	↓ *
l-Dopachrome	0.90	232.118	[M + K]^+^	↓ ***	↓ *
Inosinic acid	2.06	371.1956	[M + Na]^+^	↓ ***	↓ *
NADH	8.61	664.432	[M + H]^+^	↓ ***	↓ *
Sorbitol	3.25	200.2009	[M + NH_4_]^+^	↓ *	↓ **
5′-Deoxy-5-fluorocytidine	8.18	263.237	[M + Na]^+^	↓ *	↓ **
Iodotyrosine	1.25	330.0728	[M + Na]^+^	↓ **	↓ **
l-3-Hydroxykynurenine	8.08	242.2477	[M + NH4]^+^	↓ **	↓ **
Maleylacetoacetic acid	0.57	239.1142	[M + K]^+^	↓ ***	↓ **
Dihydroxyacetone Phosphate Acyl Ester	1.40	199.072	[M + H]^+^	↓ ***	↓ **
Glyceric acid	4.05	107.0858	[M + H]^+^	↓ ***	↓ **
Isoprenyl alcohol	4.05	125.0963	[M + K]^+^	↓ ***	↓ **
4-Aminobutyraldehyde	4.56	110.105	[M + Na]^+^	↓ ***	↓ **
Dihydrouracil	4.56	137.0963	[M + Na]^+^	↓ ***	↓ **
Normetanephrine	4.69	206.1903	[M + Na]^+^	↓ ***	↓ **
Topaquinone	6.42	234.1598	[M + Na − 2H]^−^	↓ ***	↓ **
l-Cystathionine	7.43	261.2212	[M + K]^+^	↓ ***	↓ **
N,N′-Diacetylhydrazine	4.61	139.1118	[M + Na]^+^	↓ *	↓ ***
4-Hydroxyphenylpyruvic acid	4.05	198.1855	[M + NH_4_]^+^	↓ **	↓ ***
Indoxyl sulfate	7.12	214.2167	[M + H]^+^	↓ **	↓ ***
cis-Aconitic acid	0.64	175.1196	[M + H]^+^	↓ ***	↓ ***
Cyclic pyranopterin monophosphate	1.40	386.214	[M + Na]^+^	↓ ***	↓ ***
Guanosine 3′-diphosphate 5′-triphosphate	1.63	684.1546	[M + H]^+^	↓ ***	↓ ***
Dihydroxyacetone phosphate	2.89	193.0497	[M + Na]^+^	↓ ***	↓ ***
Indolepyruvate	4.03	226.1775	[M + Na]^+^	↓ ***	↓ ***
2-Hydroxy-3-(4-hydroxyphenyl)propenoic acid	4.05	181.1592	[M + H]^+^	↓ ***	↓ ***
Glucosamine	4.05	180.1748	[M + H]^+^	↓ ***	↓ ***
5-Methoxyindoleacetate	4.56	228.1958	[M + Na]^+^	↓ ***	↓ ***
Phosphohydroxypyruvic acid	4.71	207.0327	[M + Na]^+^	↓ ***	↓ ***
5-Acetylamino-6-formylamino-3-methyluracil	6.70	249.1845	[M + Na]^+^	↓ ***	↓ ***
7-Methyluric acid	7.35	205.1231	[M + Na]^+^	↓ ***	↓ ***
N-Glycolylneuraminic acid	8.56	326.2705	[M + H]^+^	↓ ***	↓ ***

↑: up-regulated, ↓: down-regulated; *, *p* < 0.05, **, *p* < 0.01, ***, *p* < 0.001; ns: no significant differences.

**Table 3:** Differential metabolites involvement in different pathways.

Pathway	NDMF	NDMM	Pathway	NDMF	NDMM
Tyrosine metabolism	8	6	Alanine, aspartate and glutamate metabolism	1	1
Glycine, serine and threonine metabolism	3	5	beta-Alanine metabolism	1	1
Tryptophan metabolism	4	3	Citrate cycle (TCA cycle)	1	1
Cysteine and methionine metabolism	2	3	Folate biosynthesis	1	1
Amino sugar and nucleotide sugar metabolism	3	2	Inositol phosphate metabolism	1	1
Fructose and mannose metabolism	3	2	Nicotinate and nicotinamide metabolism	1	1
Glycerolipid metabolism	3	2	Pantothenate and CoA biosynthesis	1	1
Glycerophospholipid metabolism	3	2	Phenylalanine, tyrosine and tryptophan biosynthesis	1	1
Pentose phosphate pathway	3	2	Ubiquinone and other terpenoid-quinone biosynthesis	1	1
Caffeine metabolism	2	2	Vitamin B6 metabolism	1	1
Drug metabolism–other enzymes	2	2	Pentose and glucuronate interconversions	0	1
Ether lipid metabolism	2	2	Pyruvate metabolism	0	1
Glyoxylate and dicarboxylate metabolism	2	2	Histidine metabolism	1	0
Purine metabolism	2	2	Metabolism of xenobiotics by cytochrome P450	1	0
Glycolysis/Gluconeogenesis	1	2	Riboflavin metabolism	1	0
Arginine and proline metabolism	2	1	Valine, leucine and isoleucine biosynthesis	1	0
Galactose metabolism	2	1	Valine, leucine and isoleucine degradation	1	0
Pyrimidine metabolism	2	1			

NDMF: number of differential metabolites in females, NDMM: number of differential metabolites in males.

## Data Availability

The original contributions presented in this study are included in the article. Further inquiries can be directed to the corresponding author.
